# Effect of simulated microgravity on the antidiabetic properties of wheatgrass (*Triticum aestivum*) in streptozotocin-induced diabetic rats

**DOI:** 10.1038/s41526-020-0096-x

**Published:** 2020-02-24

**Authors:** Wajdy J. Al-Awaida, Ahmad S. Sharab, Hamzeh J. Al-Ameer, Nabil Y. Ayoub

**Affiliations:** 1 Department of Biology and Biotechnology, American University of Madaba, Madaba, Jordan; 2 Department of Basic Sciences and Humanities, Faculty of Science, American University of Madaba (AUM), Amman, 11821 Jordan

**Keywords:** Medicinal chemistry, Type 1 diabetes, Type 1 diabetes, Medicinal chemistry

## Abstract

Microgravity affects plant growth and content. A three-dimensional clinostat was used at 4 rotations/min to rotate the seeds of *Triticum aestivum* cultivar (Ammon) in three dimensions for 7 days, following which the antioxidant activities of ethanolic extracts were evaluated using both nitric oxide- and hydrogen peroxide-scavenging activities. The antidiabetic activities of ethanolic extracts were evaluated by measuring the concentration of plasma glucose, insulin, C peptide, and glycated hemoglobin (HbA1c); determining the number of β cells in the pancreatic islets; and performing the glucose tolerance test. Furthermore, the effects of the ethanolic extracts on the lipid profile and liver function were estimated. After rats were sacrificed, their pancreases were isolated and used for histopathological processing. The results indicated that the antioxidant potential and antioxidant metabolite content were significantly increased under microgravity conditions in comparison to those under normal gravity conditions. Rats treated with an extract of wheatgrass (*T. aestivum*) germinated over a period of 6–10 days under microgravity (WGM) showed a significant reduction in the levels of serum glucose, HbA1C, urea, creatinine, aspartate aminotransferase and alanine aminotransferase, and insulin resistance compared to rats treated with an extract of wheatgrass germinated under gravity. Additionally, the total cholesterol and low-density lipoprotein cholesterol levels were significantly decreased. In contrast, high-density lipoprotein cholesterol, C-peptide, and insulin levels rose significantly after treatment with *T. aestivum* germinated under microgravity. WGM is a promising potential diabetic treatment without side effects with a low manufacturing cost.

## Introduction

Diabetes is a metabolic syndrome that arises mainly due to deficiencies in insulin activity, insulin secretion, or both.^1^ This disorder can cause serious problems that affect human health.^2^ Over the long term, uncontrolled diabetes can lead to several chronic complications, including renal failure, heart disease, and blindness.^3^ Statistically, 8.8% of the world’s population in 2015 exhibited the symptoms of this disease, and this percentage is predicted to rise to more than 10.4% by 2040.^4^

Despite the significant developments made in the management of diabetes over the past two decades, the effects of diabetes treatments in patients are still far from exemplary.^5^ These treatments have many disadvantages, including a reduction in drug efficiency, toxicity, and side effects. For example, sulfonylureas lose their efficiency after 6 years of treatment in 44% of patients. It is also believed that glucose-lowering medications are not capable of controlling hyperlipidemia.^6^ Recently, the use of herbal medications with insignificant toxicity and no side effects for the treatment of diabetes mellitus has been demonstrated to be of global importance.^7^

Wheatgrass is believed to have a better nutritive value than nongerminated grains and their products.^8^ Antioxidant compounds, such as vitamins C, flavonoids, and phenolic compounds, are scarcely measurable in dry grains. However, upon germination, the concentrations of these antioxidant compounds increase with increasing germination time and peak after 7 days.^9^

There are numerous hypoglycemic medications available in the clinic, but these medications are associated with many side effects. Therefore, the current focus on diabetes treatment is on the development of an alternative solution for the treatment of diabetes from natural sources, which are affordable and effective and have fewer side effects than synthetic drugs.^10^ In traditional medicine, medicinal plants are used for the treatment of diabetes; these plants are a rich source of hypolipidemic, hypoglycemic, and antioxidant agents, such as phenols, flavonoids, gallotannins, and other related polyphenols.^11,12^

Wheatgrass (*T. aestivum* germinated over a period of 6–10 days) was found to improve metabolic and lipid profiles^13^ and to restore the levels of plasma glucose, insulin, and liver glycogen.^14,15^

The nutrient value of germinated grains depends on the conditions in which they are grown. These conditions include humidity, temperature, length of germination, culture medium, and steeping protocol.^16^ The microgravity environment encountered during space flight has long been thought to affect plant growth and content.^17^ Until now, no research on the therapeutic potential of wheatgrass germinated under microgravity (WGM) conditions has been carried out. Therefore, the present study was carried out to produce wheatgrass with high antioxidant and antidiabetic potential, but no side effects with a low manufacturing cost.

## Results

**Fig. 1:**
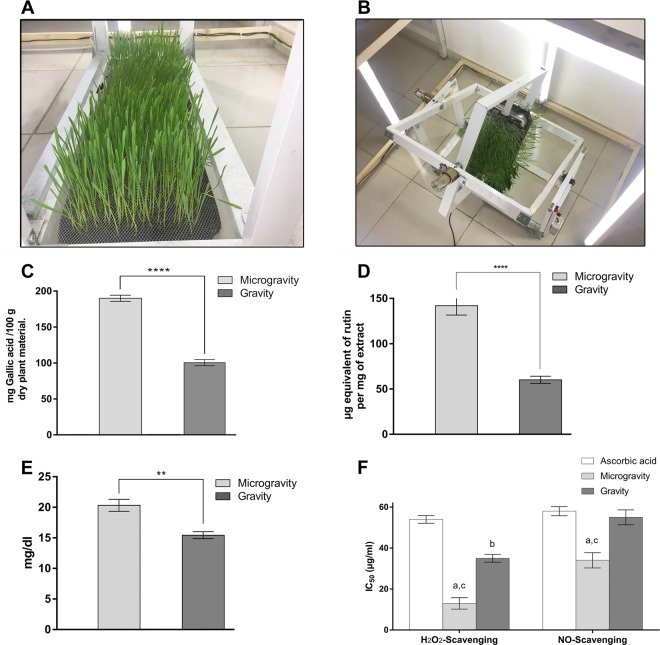
*Triticum aestivum* growth under gravity and microgravity conditions and the effect of simulated microgravity on the total phenolic content, total flavonoid content, vitamin C content, and total antioxidant activities in wheatgrass extract. **a** shows *T. aestivum* growth under gravity conditions. **b** shows *T. aestivum* growth under microgravity conditions simulated by a three-dimensional clinostat. **c** shows the total phenolic content of ethanolic extracts of *T. aestivum* under gravity and microgravity conditions. **d** shows the total flavonoid content of ethanolic extracts of *T. aestivum* under gravity and microgravity conditions. **e** shows the vitamin C content of ethanolic extracts of *T. aestivum* under gravity and microgravity conditions. **f** shows the hydrogen peroxide- and nitric oxide radical-scavenging activities of ethanolic extracts of *T. aestivum* under gravity and microgravity conditions. The values are expressed as the mean ± SEM of seven rats in each group. Data in **c**–**e** were analyzed by using two-way ANOVA, followed by Tukey’s multiple comparison test with the level of significance set at *P* < 0.05. *, **, ***, **** = *P* < 0.05, *P* < 0.01, *P* < 0.001, and *P* < 0.0001, respectively. Data in **f** were analyzed by using two-way ANOVA, followed by Tukey’s multiple comparison test with the level of significance set at *P* < 0.05. a refers to microgravity versus a reference compound ascorbic acid; b refers to gravity versus a reference compound ascorbic acid; and c refers to microgravity versus a gravity.

Plant phenolics and flavonoids play a substantial role in scavenging free radicals in the body and act as antioxidants. Our results showed the total phenolic content (TPC) of the ethanolic extracts of wheatgrass germinated under gravity (WGG) and WGM conditions. The TPC is expressed as mmol equivalents of gallic acid/100 g fresh weight. In the ethanolic extract of WGM, the TPC (190.0 ± 2.11 mmol) was found to be significantly higher (*P* < 0.0001; TPC of extracts by two-way analysis of variance (ANOVA), followed by Tukey’s multiple comparison test) than that of the ethanolic extract of wheatgrass grown under gravity (100.4 ± 2.15 mmol), as shown in Fig. 1c.

Our results present the total flavonoid content (TFC) of ethanolic extracts of WGG and WGM conditions. The TFC is expressed as mmol equivalents of rutin/mg fresh weight. The TFC of the ethanolic extract of WGM was significantly higher (208.1 ± 6.57 mmol) (*P* < 0.0001; TFC of extracts by two-way ANOVA followed by Tukey’s multiple comparison test) than the TFC of the ethanolic extract of WGG (30.68 ± 4.01 mmol), as shown in Fig. 1d.

Vitamin C, also known as ascorbic acid, is an antioxidant compound and a component of an enzyme required for protein metabolism. Vitamin C also helps with iron absorption and is important for immune system health.

The quantity of vitamin C, quantified as the total ascorbic acid content, was higher in the ethanolic extract of WGM (20.32 ± 0.56 mg/dl) (*P* < 0.01; vitamin C of extracts by two-way ANOVA, followed by Tukey’s multiple comparison test) than in the ethanolic extract of WGG (15.43 ± 0.32 mg/dl) (Fig. 1e).

Hydrogen peroxide and nitric oxide radical-scavenging activities were measured to determine the ability of the WGM extracts to detoxify and scavenge free radicals.

Our results present the hydrogen peroxide and nitric oxide radical-scavenging activities of ethanolic extracts of WGG and WGM. The ethanolic extract of WGM showed significantly greater (*P* < 0.05; H_2_O_2_-scavenging activity of extracts by two-way ANOVA, followed by Tukey’s multiple comparison test) H_2_O_2_-scavenging activity with a lower half-maximal inhibitory concentration (IC_50_) value (13.0 ± 2.8 µg/ml) compared to ascorbic acid (54.9 ± 1.90 µg/ml), which was used as a positive control, and the ethanolic extract of WGG (35.0 ± 1.93 µg/ml). In contrast, the ethanolic extract of WGM showed significantly greater (*P* < 0.05; nitric oxide-scavenging activity of extracts by two-way ANOVA, followed by Tukey’s multiple comparison test) nitric oxide-scavenging activity with a lower IC_50_ compared to the positive control, ascorbic acid, and the ethanolic extract of WGG, as shown in Fig. 1f.

**Fig. 2:**
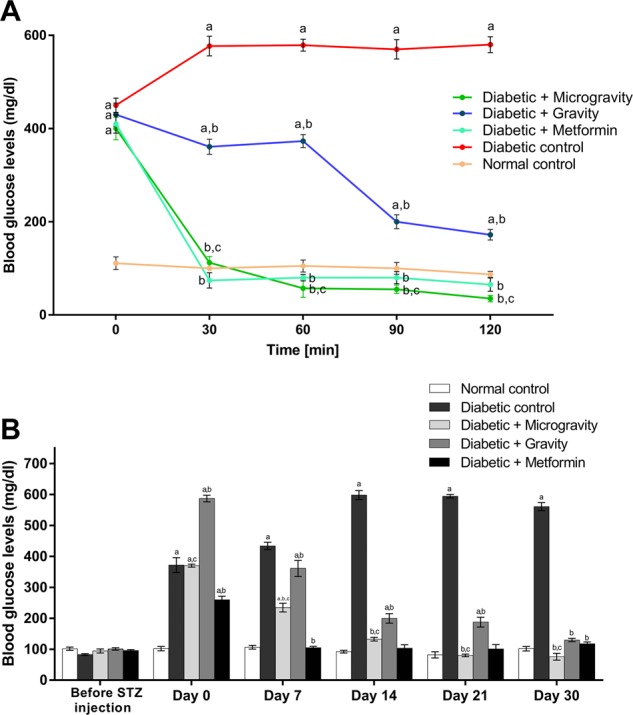
The effects of ethanolic extracts of *T. aestivum* germinated under gravity and microgravity conditions on oral glucose tolerance tests over 2 h and fasting blood glucose levels over 30 days in experimental rats. **a** shows the effect of ethanolic extracts of *T. aestivum* under gravity and microgravity conditions on the results of the oral glucose tolerance test (OGTT). **b** shows the effect of ethanolic extracts of *T. aestivum* under gravity and microgravity conditions on fasting blood glucose levels in experimental rats. The values are expressed as the mean ± SEM of seven rats in each group. Data were analyzed by using two-way ANOVA followed by Tukey’s multiple comparison test with the level of significance set at *P* < 0.05. a refers to treatment versus normal control; b refers to treatment versus diabetic control; c refers to microgravity versus gravity.

The glucose tolerance test (GTT) has been extensively used for the study of carbohydrate metabolism in experimental animals. Time-dependent changes in blood glucose levels during the oral GTT (0–120 min) for all groups are shown in Fig. 2a. The rats in the diabetic group had significantly higher (*P* < 0.05; GTT by two-way ANOVA, followed by Tukey’s multiple comparison test) blood glucose levels throughout the total experimental period (120 min) (450.0 ± 15.14 to 580.0 ± 17.07 mg/dl) compared to the normal control group (111.0 ± 13.0 to 87.0 ± 13.0 mg/dl) (Fig. 2a). In diabetic rats treated with the ethanolic extract of WGG, even after the administration of glucose, blood glucose levels remained the same as those at 0 min (430 ± 24.74 mg/dl) and even those at 60 min (373 ± 14.14 mg/dl), following which the blood glucose levels began to decrease after 90 min (200.0 ± 14.84 mg/dl) and reached the upper limit for normal glucose levels after 120 min (172.0 ± 11.31 mg/dl) (Fig. 2a, *P* < 0.05; GTT by two-way ANOVA, followed by Tukey’s multiple comparison test). In diabetic rats treated with the ethanolic extract of WGM, the blood glucose levels began to decrease, reached the control level after 30 min (112.0 ± 13.36 mg/dl) and remained the same until 120 min (105.0 ± 7.0 mg/dl) (Fig. 2a). In diabetic rats treated with metformin, blood glucose levels began to decrease and reached the control level after 30 min (74.0 ± 16.36 mg/dl), at which they remained until 120 min (65.0 ± 14.36 mg/dl) (Fig. 2a). In the diabetic group treated with the ethanolic extract of WGM, the blood glucose levels were significantly decreased compared to those in the diabetic control group, the diabetic group treated with the ethanolic extract of WGG, and the diabetic group treated with metformin (Fig. 2a).

Diabetes is characterized by a high level of glucose in the bloodstream caused by insufficient insulin secretion or insulin resistance.

The diabetic group showed a significant increase (*P* < 0.05; blood glucose levels by two-way ANOVA, followed by Tukey’s multiple comparison test) in blood glucose levels in comparison to the control group. First, oral administration of the ethanolic extract of WGM in the diabetic group revealed a highly significant reduction (*P* < 0.05; blood glucose levels by two-way ANOVA, followed by Tukey’s multiple comparison test) in blood glucose levels starting on the seventh day of treatment (234.50 ± 14.28 mg/dl) compared to the levels in the same group before treatment (day 0) (370.0 ± 4.81 mg/dl) as well as the levels in the diabetic group on the corresponding days (560.0 ± 13.06 and 371.50 ± 24.11 mg/dl, respectively). This reduction was marked on the 30th day, as blood glucose levels were reduced by 79.5% compared to those at day 0 and 84.7% compared to the value in the diabetic group (Fig. 2b).

Subsequently, the group treated with the ethanolic extract of WGG exhibited a highly significant reduction (*P* < 0.05; blood glucose levels by two-way ANOVA, followed by Tukey’s multiple comparison test) in blood glucose levels starting on the seventh day of treatment (361.0 ± 25.92 mg/dl) compared to levels on day 0 (587.0 ± 10.61 mg/dl) and the levels in diabetic rats on the corresponding days (560.0 ± 13.06 and 371.50 ± 24.11 mg/dl, respectively). This reduction was marked on the 30th day, as blood glucose levels were reduced by 77.9% compared to those on day 0 and 76.8% compared to those in the diabetic group (Fig. 2b). The diabetic group treated with metformin showed a significant reduction (*P* < 0.05; blood glucose levels by two-way ANOVA, followed by Tukey’s multiple comparison test) in blood glucose levels starting on the seventh day of treatment (105.0 ± 4.08 mg/dl) compared to blood glucose levels on day 0 (259.50 ± 11.83 mg/dl) and those of the diabetic group on the corresponding days (560.0 ± 13.06 and 371.50 ± 24.11 mg/dl, respectively). This reduction was marked on the 30th day, as blood glucose levels were reduced by 54.8% compared to those on day 0 and 79.1% compared to those in the diabetic group (Fig. 2b). In the diabetic group treated with the ethanolic extract of WGM, blood glucose levels were significantly decreased (*P* < 0.05; blood glucose levels by two-way ANOVA, followed by Tukey’s multiple comparison test) compared to the group treated with the ethanolic extract of WGG beginning on day 7 (234.50 ± 14.28 and 361.0 ± 25.92 mg/dl, respectively). This reduction was marked on the 30th day (75.50 ± 11.02 and 129.50 ± 5.30 mg/dl), when blood glucose were 41.6% lower in the group treated with the ethanolic extract of WGM (Fig. 2b).

**Fig. 3:**
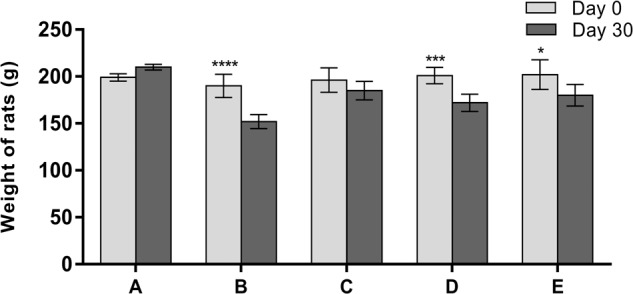
The effects of ethanolic extracts of *T. aestivum* germinated under gravity and microgravity conditions on body weight in experimental rats after 30 days. **a** shows changes in body weight in untreated nondiabetic rats on days 0 and 30. **b** shows changes in body weight in untreated diabetic rats on days 0 and 30. **c** shows changes in diabetic rats treated with the ethanolic extract of *T. aestivum* germinated under microgravity conditions on days 0 and 30. **d** shows changes in diabetic rats treated with the ethanolic extract of *T. aestivum* germinated under gravity condition group on days 0 and 30. **e** shows changes in diabetic rats treated with metformin on days 0 and 30. All values represent the mean ± SD. All comparisons were performed between the same group of animals on days 0 and 30. Data were analyzed by using two-way ANOVA, followed by Tukey’s multiple comparison test with the level of significance set at *P* < 0.05. *, ***, **** = *P* < 0.05,  , *P* < 0.001, and *P* < 0.0001, respectively.

Insufficient insulin prevents the body from transporting glucose from the bloodstream into the body’s cells to use as energy. When this occurs, the body starts burning fat and muscle for energy, causing a decrease in overall body weight. Changes in the body weight of rats in the normal control, diabetic control, microgravity, gravity, and metformin groups over a period of 30 days are shown in Fig. 3. A significant decrease (*P* < 0.05; body weight by two-way ANOVA, followed by Tukey’s multiple comparison test. *, **, ***, **** = *P* < 0.05, *P* < 0.01, *P* < 0.001, and *P* < 0.0001, respectively) in body weight was observed in the diabetic and gravity groups compared with their body weights at the initial day of the experiment (from 190.0 ± 12.4 to 152.0 ± 7.49 g and from 201.0 ± 8.7 to 172.0 ± 9.23 g, respectively). However, there was no significant difference (*P* < 0.05; body weight by two-way ANOVA, followed by Tukey’s multiple comparison test. *, **, ***, **** = *P* < 0.05, *P* < 0.01, *P* < 0.001, and *P* < 0.0001, respectively) in body weight between the normal control, microgravity, and metformin groups on the initial day of the experiment (Fig. 3).

Glycated hemoglobin (HbA1c), a form of hemoglobin that is chemically linked to glucose, indicates the sugar level in the bloodstream over a long period.

**Fig. 4:**
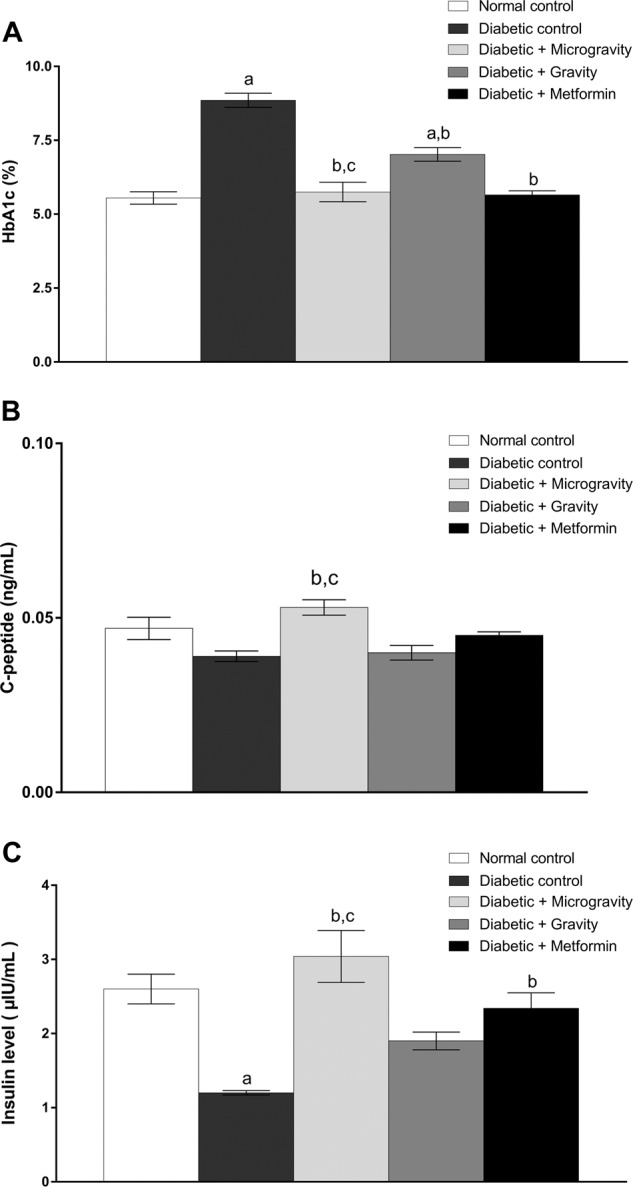
Effect of treatment with ethanolic extracts of *T. aestivum* under gravity and microgravity conditions for 30 days on C-peptide, serum insulin, and glycated hemoglobin (HbA1c) levels in experimental rats. **a** shows the effect of ethanolic extracts of *T. aestivum* germinated under gravity and microgravity conditions on body weight in experimental rats. **b** shows the effect of ethanolic extracts of *T. aestivum* germinated under gravity and microgravity conditions on C-peptide levels in experimental rats. **c** shows the effect of ethanolic extracts of *T. aestivum* germinated under gravity and microgravity conditions on serum insulin levels in experimental rats. The values are expressed as the mean ± SEM of seven rats in each group. Data were analyzed by using two-way ANOVA, followed by Tukey’s multiple comparison test with the level of significance set at *P* < 0.05. a refers to treatment versus normal control, b refers to treatment versus diabetic control, and c refers to microgravity versus gravity.

Our results show the HbA1C levels in normal and experimental rats. In the diabetic control group, there was a significant increase (*P* < 0.05; HbA1C level by two-way ANOVA, followed by Tukey’s multiple comparison test) in the HbA1C level (8.85 ± 0.24%) compared to the normal control group (5.55 ± 0.21%) (Fig. 4a). In the diabetic group treated with the ethanolic extract of WGM, the HbA1C level was significantly decreased (5.75 ± 0.33%) (*P* < 0.05; HbA1C level by two-way ANOVA, followed by Tukey’s multiple comparison test) compared to the diabetic control group (Fig. 4a). In the diabetic group treated with the ethanolic extract of WGG, the HbA1C levels was significantly decreased (7.02 ± 0.23%) (*P* < 0.05; HbA1C level by two-way ANOVA, followed by Tukey’s multiple comparison test) compared to the diabetic group. The HbA1C level was higher in the diabetic gravity group. In diabetic rats treated with metformin, the HbA1C levels was significantly decreased (5.65 ± 0.14%) (*P* < 0.05; HbA1C level by two-way ANOVA, followed by Tukey’s multiple comparison test) compared to the diabetic group. A significant difference (*P* < 0.05; HbA1C level by two-way ANOVA, followed by Tukey’s multiple comparison test) in the HbA1C level was observed between the gravity and WGM groups. The HbA1C level in the diabetic group treated with WGM was significantly lower than the HbA1C level in the diabetic group treated with WGG (Fig. 4a).

C-peptide of insulin, a metabolic byproduct produced during the activation of insulin, is a useful and common method used to assess pancreatic β cell function.

The C-peptide level was reduced in the diabetic group (0.039 ± 0.0015 ng/ml) compared to the normal group (0.047 ± 0.0032 ng/ml) (Fig. 4b). In the diabetic group treated with the ethanolic extract of WGM, the C-peptide level was significantly increased (0.053 ± 0.0022 ng/ml (*P* < 0.05; C-peptide level by two-way ANOVA, followed by Tukey’s multiple comparison test) compared to the diabetic group by 1.35-fold (Fig. 4b). There was no significant difference (*P* < 0.05; C-peptide level by two-way ANOVA, followed by Tukey’s multiple comparison test) in the C-peptide level between the diabetic group treated with the ethanolic extract of WGG (0.04 ± 0.0021 ng/ml) and both the diabetic and normal control groups (Fig. 4b). C-peptide levels in the diabetic group treated with metformin (0.045 ± 0.0010 ng/ml) were not significantly different (*P* < 0.05; C-peptide level by two-way ANOVA, followed by Tukey’s multiple comparison test) compared to those in the diabetic group (Fig. 4b). In the diabetic group treated with the ethanolic extract of WGG, there was a significant increase (*P* < 0.05; C-peptide level by two-way ANOVA, followed by Tukey’s multiple comparison test) in the C-peptide level insulin level by 1.32-fold compared to the gravity group (Fig. 4b).

The plasma insulin level was significantly reduced (*P* < 0.05; insulin level by two-way ANOVA, followed by Tukey’s multiple comparison test) in the diabetic group (1.2 ± 0.30 µlU/ml) compared to the normal control group (2.6 ± 0.20 µlU/ml) (Fig. 4c). The plasma insulin level in the diabetic group treated with the ethanolic extract of WGM was significantly increased by 2.53-fold (3.04 ± 0.35 µlU/ml) (*P* < 0.05; insulin level by two-way ANOVA, followed by Tukey’s multiple comparison test) compared to the diabetic control group (Fig. 4c). There was no significant difference (*P* < 0.05; insulin level by two-way ANOVA, followed by Tukey’s multiple comparison test) in the plasma insulin level of the diabetic group treated with the ethanolic extract of WGG (1.9 ± 0.12 µlU/ml) compared to the diabetic and normal control groups (Fig. 4c). In diabetic rats treated with metformin, the insulin level was significantly increased (2.34 ± 0.21 µlU/ml) (*P* < 0.05; insulin level by two-way ANOVA, followed by Tukey’s multiple comparison test) by 1.95-fold compared to the diabetic group (Fig. 4c). In the diabetic group treated with the ethanolic extract of WGM, the insulin level was significantly increased (*P* < 0.05; insulin level by two-way ANOVA, followed by Tukey’s multiple comparison test) by 1.6-fold compared to the gravity group (Fig. 4c).

**Fig. 5:**
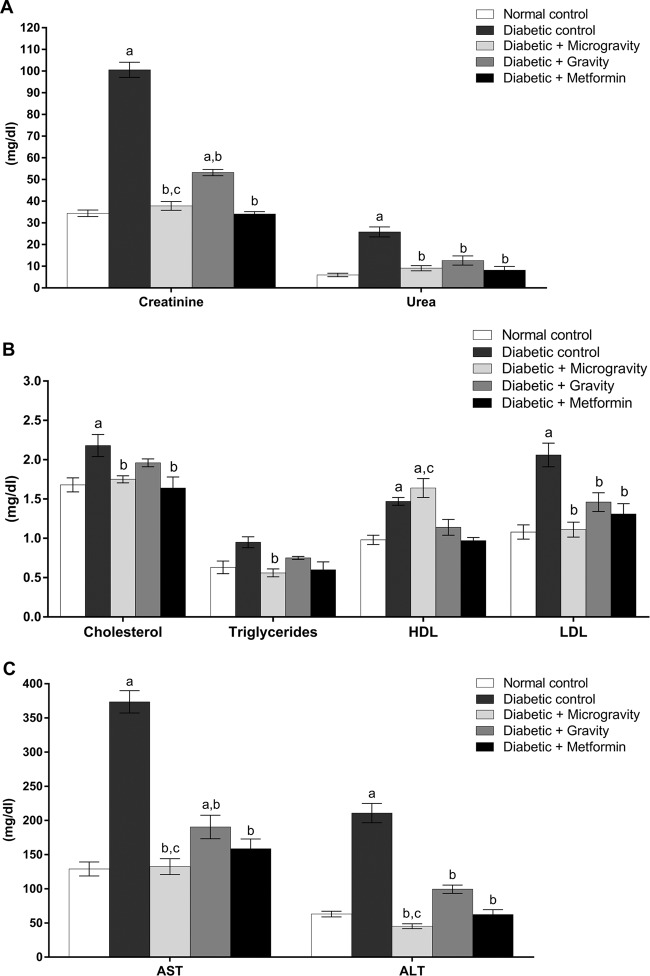
Effect of treatment with ethanolic extracts of *T. aestivum* under gravity and microgravity conditions for 30 days on liver and kidney functions and lipid profiles in experimental rats. **a** shows the effect of ethanolic extracts of *T. aestivum* under gravity and microgravity conditions on biomarkers of kidney function in experimental rats. **b** shows the effect of ethanolic extracts of *T. aestivum* under gravity and microgravity conditions on the lipid profiles of experimental rats. **c** shows the effect of ethanolic extracts of *T. aestivum* under gravity and microgravity conditions on biomarkers of liver function in experimental rats. The values are expressed as the mean ± SEM of seven rats in each group. Data were analyzed by using two-way ANOVA, followed by Tukey’s multiple comparison test with the level of significance set at *P* < 0.05. a refers to treatment versus normal control, b refers to treatment versus diabetic control, and c refers to microgravity versus gravity.

Serum urea and creatinine are known to be increased with hyperglycemia in patients with uncontrolled diabetes and usually correlate with the severity of kidney damage. Our results show the levels of creatinine and urea in normal and experimental rats. In diabetic rats, creatinine and urea levels were significantly increased (100.60 ± 3.50 and 25.80 ± 2.30 mg/dl, respectively) (*P* < 0.05; creatinine and urea levels by two-way ANOVA, followed by Tukey’s multiple comparison test) compared to the normal control group (34.40 ± 1.51 and 6.0 ± 0.80 mg/dl, respectively) (Fig. 5a). The creatinine level in the diabetic group treated with the ethanolic extract of WGM was significantly decreased (37.80 ± 2.03 mg/dl) (*P* < 0.05; creatinine level by two-way ANOVA, followed by Tukey’s multiple comparison test) compared to the diabetic control group (100.60 ± 3.50 mg/dl) (Fig. 5a). The creatinine level in the diabetic group treated with the ethanolic extract of WGG was significantly decreased (53.20 ± 1.43 mg/dl) (*P* < 0.05; creatinine level by two-way ANOVA, followed by Tukey’s multiple comparison test) compared to the diabetic control group (100.60 ± 3.50 mg/dl) (Fig. 5a). The creatinine level in the diabetic gravity group was higher than that in the diabetic microgravity group. In diabetic rats treated with metformin, the creatinine level was significantly increased (34.10 ± 1.08 mg/dl) (*P* < 0.05; creatinine level by two-way ANOVA, followed by Tukey’s multiple comparison test) compared to the diabetic control group (100.60 ± 3.50 mg/dl) (Fig. 5a). The blood urea level was increased in the diabetic group (25.8 ± 2.30 mg/dl). However, the blood urea level was significantly decreased (*P* < 0.05; urea level by two-way ANOVA, followed by Tukey’s multiple comparison test) in the microgravity (9.10 ± 1.20 mg/dl), gravity (12.63 ± 2.10 mg/dl) and metformin (8.20 ± 1.70 mg/dl) groups compared to the diabetic control group. No significant differences (*P* < 0.05; urea level by two-way ANOVA, followed by Tukey’s multiple comparison test) in blood urea level were found in the microgravity, gravity, and metformin groups in comparison to the normal control group (Fig. 5a).

Uncontrolled diabetes tends to raise total cholesterol (TC), total triglyceride (TG), and low-density lipoprotein cholesterol (LDL-C) levels, which increases the risk of heart disease and stroke. This common condition is called diabetic dyslipidemia.

Diabetic rats showed a significant increase (*P* < 0.05; TC, TG, and LDL-C levels by two-way ANOVA followed by Tukey’s multiple comparison test) in the TC (2.18 ± 0.14 mg/dl), TG (0.85 ± .17 mg/dl), and LDL-C (2.06 ± 0.15 mg/dl) levels in the blood (Fig. 5b). In contrast, the high-density lipoprotein cholesterol (HDL-C) level (1.47 ± 0.05 mg/dl) was significantly reduced (*P* < 0.05; HDL-C level by two-way ANOVA, followed by Tukey’s multiple comparison test) compared to the normal control group. In diabetic rats treated with the ethanolic extract of WGM, the TC (1.75 ± 0.04 mg/dl), TG (0.56 ± 0.05 mg/dl), and LDL-C (1.11 ± 0.9 mg/dl) levels in the blood were significantly decreased (*P* < 0.05; TC, TG, and LDL-C levels by two-way ANOVA, followed by Tukey’s multiple comparison test). However, the level of HDL-C (1.64 ± 0.12 mg/dl) was significantly increased (*P* < 0.05; HDL-C level by two-way ANOVA, followed by Tukey’s multiple comparison test) (Fig. 5b). In diabetic rats treated with the ethanolic extract of WGG, the blood level of LDL-C (1.46 ± 0.12 mg/dl) was significantly decreased (*P* < 0.05; LDL-C level by two-way ANOVA, followed by Tukey’s multiple comparison test). However, TC (1.96 ± .05 mg/dl), HDL-C (1.14 ± 0.10 mg/dl), and TG (0.75 ± 0.20 mg/dl) levels were not changed (Fig. 5b). In diabetic rats treated with metformin, the TC (1.64 ± 0.14 mg/dl) and LDL-C (1.31 ± 0.13 mg/dl) levels in the blood were significantly decreased (*P* < 0.05; TC and LDL-C levels by two-way ANOVA, followed by Tukey’s multiple comparison test). However, the TG (0.60 ± 0.10 mg/dl) and HDL-C (0.97 ± 0.04 mg/dl) levels were not changed (Fig. 5b). The level of HDL-C in the microgravity group was significantly increased (*P* < 0.05; HDL-C level by two-way ANOVA, followed by Tukey’s multiple comparison test) compared to the gravity group (Fig. 5b).

Increased activities of the liver enzymes aspartate aminotransferase (AST) and alanine aminotransferase (ALT) are indicators of liver cell injury and associated with uncontrolled diabetes.

The induction of diabetes led to significant increases (*P* < 0.05; AST and ALT levels by two-way ANOVA, followed by Tukey’s multiple comparison test) in the serum activities of AST (373.50 ± 16.30 mg/dl) and ALT (210.70 ± 14.10 mg/dl) in the diabetic group compared to the normal group (129.0 ± 10.20 and 63.0 ± 4.20 mg/dl, respectively) (Fig. 5c). ALT (132.40 ± 11.60 mg/dl) and AST (45.20 ± 3.60 mg/dl) activities were significantly reduced (*P* < 0.05; AST and ALT levels by two-way ANOVA, followed by Tukey’s multiple comparison test) in the group of diabetic rats treated with the ethanolic extract of WGM compared to the diabetic group. The AST and ALT levels in the diabetic gravity group were higher than those in the diabetic microgravity group. (Fig. 5c). Interestingly, the diabetic group treated with metformin showed a significant reduction (*P* < 0.05; AST and ALT levels by two-way ANOVA, followed by Tukey’s multiple comparison test) in ALT (158.0 ± 14.30 mg/dl) and AST (62.30 ± 7.20 mg/dl) activities compared to the diabetic group (Fig. 5c).

β Cells are a type of cell found in pancreatic islets that produce and secrete insulin. In diabetes, β cell mass and function are diminished, leading to insufficient insulin secretion and hyperglycemia.

**Fig. 6:**
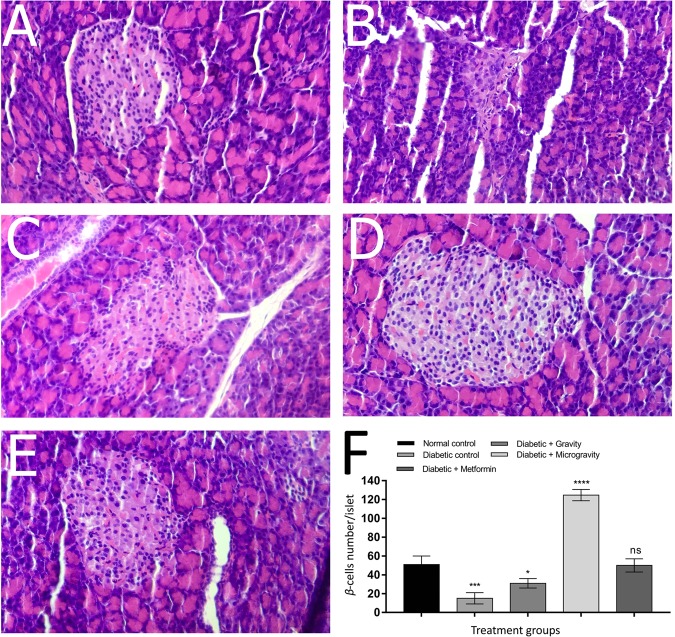
Photomicrographs of H&E-stained histological slides of pancreatic specimens. **a** Normal histological appearance of the islets of Langerhans from the pancreases of rats in the control group. **b** The pancreases of rats in the diabetic control group showed degeneration, necrotic changes, and islet shrinkage. **c** The appearance of pancreas sections from rats administered the ethanolic extract of *T. aestivum* under gravity conditions showed improved islet morphology. **d** The appearance of pancreas sections from rats administered the ethanolic extract of *T. aestivum* under microgravity conditions showed the normal structure of the pancreatic islets of Langerhans. **e** shows that there were no pathological changes in the islets of Langerhans of the pancreas in diabetic rats treated with metformin. Original magnification was ×400. **f** The pancreatic β cell number per islet in different treatment groups. Groups were compared to the control untreated group. As shown in the figure, microgravity extract treatment had a highly significant effect on the pancreatic β cell number per islet compared to the normal untreated control group. Data were compared against data for the normal control by using one-way ANOVA with Bonferroni’s multiple comparisons test with the level of significance at *P* < 0.05. *, ***, ****, ns = *P* < 0.05, *P* < 0.001, *P* < 0.0001, and nonsignificant, respectively.

To elucidate the preventative effects of ethanolic extracts of WGG and WGM conditions on streptozotocin (STZ)-induced diabetes, the pancreatic tissues of the ethanol extract-treated and control groups were examined. The results revealed the following: the normal control group showed a normal pancreatic structure (Fig. 6a), whereas the diabetic group showed degeneration, necrotic changes, and islet shrinkage in the pancreas (Fig. 6b). The pancreatic sections of rats administered the ethanolic extract of WGG showed improved islet morphology (Fig. 6c). The pancreatic sections of rats administered the ethanolic extract of WGM showed a normal structure in the pancreatic islets of Langerhans (Fig. 6d). There were no pathological changes in the pancreatic islets of Langerhans in diabetic rats treated with metformin (Fig. 6e). β Cells in the pancreatic islets was measured under a ×40 objective lens. The results revealed a significant decrease (*P* < 0.001; β cells by one-way ANOVA with Bonferroni’s multiple comparisons test. *, ***, ****, ns = *P* < 0.05, *P* < 0.001, *P* < 0.0001, and nonsignificant, respectively) in the number of β cells per islet in the diabetic control group (15 ± 3.46) compared to the normal control group (51 ± 5.19). In addition, there was a significant decrease (*P* < 0.05; β cells by one-way ANOVA with Bonferroni’s multiple comparisons test. *, ***, ****, ns = *P* < 0.05, *P* < 0.001, *P* < 0.0001, and nonsignificant, respectively) in the number of β cells per islet in the gravity group (31 ± 2.88) compared to the normal control group (15 ± 3.46). However, there was a significant increase (*P* < 0.0001; β cells by one-way ANOVA with Bonferroni’s multiple comparisons test. *, ***, ****, ns = *P* < 0.05, *P* < 0.001, *P* < 0.0001, and nonsignificant, respectively) in the number of β cells per islet in the microgravity group (114 ± 7.50) compared to the normal control group. However, there was no significant difference (ns) in the number of β cells per islet in the metformin group (50 ± 4.04) compared to the normal control group.

## Discussion

This study evaluated the effect of simulated microgravity on the antidiabetic and antioxidant potential of wheatgrass extracts in STZ-induced diabetic rats. Insulin-dependent diabetes mellitus (IDDM) is a disorder initiated by progressive damage to insulin-secreting β cells. STZ is normally used to induce diabetes in pancreatic rats by DNA alkylation and β cell fragmentation.^18^ STZ selectively devastates the β cells, which secrete insulin, decreasing the β cell efficiency and resulting in diabetes mellitus.^19^ Many living β cells remain after treatment with low-dose STZ, and their regeneration is also possible.^18^

In our study, significantly more β cells were observed in the group treated with the ethanol extract of WGM conditions, and stimulation due to insulin secretion could be responsible for most of the observed metabolic effects. Bioactive compounds in medicinal plants may have insulin-like properties and have been shown to enhance insulin sensitivity, improve glucose-dependent insulin secretion, and activate the regeneration of the pancreatic islets of Langerhans in STZ-induced diabetic rats. Currently, the only option for achieving normal glucose levels in diabetic patients is the stimulation of insulin secretion by medicinal plants or renewal of β cells.^20,21^ Increased free radical generation and oxidative stress together with hyperglycemia play a key role in the pathogenesis of diabetes and its late complications.^22^ β Cells are highly sensitive to destruction by oxidative stress because of their low level of free radical-scavenging enzymes, which protect the cell from the damaging effects of oxidative stress.^2^ Decreased oxidative stress due to a reduced hyperglycemic status in diabetic conditions was detected in experimental animals following the administration of antioxidant compounds.^23,24^

Although wheatgrass germinated under normal conditions (under gravity) showed significant antioxidant potential and antihyperglycemic effects in STZ-induced diabetic rats as a result of its high TP and flavonoid concentrations,^14,25^ our results under microgravity conditions show even more promise in the field. The microgravity environment created by a three-dimensional (3D) clinostat produced wheatgrass with a higher concentration of antioxidant compounds and a higher potential for antioxidant activity than WGG. We hypothesize that the presence of a high concentration of some bioactive compounds, such as phenolic compounds, flavonoids, and vitamin C, in WGM conditions produces an insulinotropic effect due to the antidiabetic effects of these compounds that facilitates a decrease in blood glucose levels. The elevated plasma glucose levels in diabetic rats were decreased due to the administration of wheatgrass germinated extract under microgravity conditions, and the administration of WGM elevated C-peptide and plasma insulin levels compared to those in diabetic control rats. The possible mechanism of action of WGM conditions is its regeneration and stimulation of β cells to release insulin, which mediates a decrease in glucose levels, as shown in our results.

Wheatgrass ethyl alcohol extract was shown to decrease mononuclear cell infiltration in the β cells of the pancreas compared to that in the untreated group. This effect coincides with the protective effect of wheatgrass extract on the diabetes parameters investigated in this study. This effect was reported using germinated *T. aestivum*.^14^

The induction of diabetes by STZ led to a reduction in body weight due to structural protein degradation as a result of disturbances in carbohydrate metabolism and thus energy metabolism.^26^ Due to damage to β cells caused by STZ, a decrease in body weight and an increase in water and food intake are commonly detected in diabetes, and these effects may be caused by metabolic changes due to the absence or deficiency of insulin.^27^ The nonsignificant changes in the body weights of diabetic rats treated with extracts of WGG and WGM conditions as well as metformin-treated diabetic rats compared to the control group revealed the effect of a normal blood glucose level, which in turn prevents the loss of body weight.

HbA1c is used as an indicator to assess the degree of hemoglobin glycation in diabetes mellitus. HbA1c was found to represent the average fasting blood glucose level of diabetic patients over the previous 120 days.^28^ Under diabetic conditions, extra glucose in the blood is covalently bound to hemoglobin to form HbA1c.^29^ Hence, HbA1c levels are increased in untreated diabetic rats. Treatment with WGM conditions significantly reduced HbA1c levels in comparison to those in the gravity and diabetic control groups.

The liver plays a central role in the metabolism, storage, excretion, and detoxification of xenobiotics and their metabolites, and AST and ALT are effective markers of liver function^30^ and STZ-induced diabetic liver injury in rats. For that reason, elevated activities of SGOT and SGPT in the plasma might be due mostly to the escape of these enzymes from the liver cell cytosol into the bloodstream, which is a signal of the hepatotoxic effect of STZ.^31^ Treatment of the diabetic rats with the extract of WGM conditions reduced the activities of these enzymes in plasma compared to those in the gravity and diabetic control groups and consequently alleviated liver damage caused by STZ-induced diabetes, as presented in our results.

However, insulin deficiency deactivates the lipoprotein lipase enzyme.^32^ Under conditions of insulin deficiency with low lipoprotein lipase, TG, cholesterol, and LDL levels were elevated, and the HDL level was decreased.^33^ This hypertriglyceridemia was reduced rapidly after insulin treatment therapy.^32^ In this study, the administration of WGM conditions significantly increased serum insulin levels, a marked decrease in TC, TG, and LDL levels in rats treated with WGM conditions and metformin-treated rats was observed in comparison to the diabetic control group. Additionally, an increased HDL level was observed compared to diabetic control rats. This activity of WGM conditions reduces hypertriglyceridemia under diabetic conditions and therefore prevents diabetic-associated complications.

Elevated plasma urea and creatinine levels signal the development of diabetic nephropathy in diabetic rats.^34^ The urea and creatinine levels in rats with diabetic nephropathy are higher than those in normal rats.^34^ The observed maintenance of urea and creatinine levels closer to those in control rats using extracts of WGM and gravity conditions, as well as metformin treatment, suggests that germinated wheatgrass extract plays an either direct or indirect primary role in protecting against diabetic nephropathy or retarding its development.

An ethyl alcohol extract was selected because of the expected presence of phenolic and flavonoid compounds with antihyperglycemic properties. The presence of phenolic and flavonoid compounds in our extract with antioxidant potential, as seen in our results, further confirms this prediction. Phenolic and flavonoid compounds were shown to have antihyperglycemic effects.^35^ Phytochemical analysis of germinated wheatgrass showed the presence of tannins, flavonoids, saponins, and sterols. Their antidiabetic effects and ability to regenerate pancreatic β cells have already been demonstrated.^36^ Sterols were shown to decrease blood sugar levels in experimental animal models.^37^ The antioxidant activity of these phenolic and flavonoid compounds depends on their redox properties, and these compounds can act as hydrogen donors, reducing agents, and singlet oxygen quenchers.^38^ Polyphenolics containing hydroxyl groups are essential plant ingredients that can defend the body against oxidative stress.^39^ The present study concluded that the microgravity environment created by a 3D clinostat produced germinated *T. aestivum* with a high concentration of antioxidant compounds and high level of potential antioxidant activity; the produced wheatgrass has potential as a diabetic treatment without any side effects and a low manufacturing cost. This method can be used to explore the therapeutic potential of WGM conditions for other diseases.

## Methods

### The 3D clinostat

Earth’s gravitational field at its surface at a certain location is a fixed vector pointing downwards (towards the Earth’s center) whose average magnitude is 9.8 m/s.^40^ Many plants and organisms contain cells that sense gravity through sedimentation due to gravity. A procedure in which cells in plants or organisms are placed in a randomizing machine in which the gravity vector loses its effect on these cells would simulate the conditions of weightlessness in outer space as the direction of the gravity vector would be lost. The rotational speed of such a machine is determined by the time scale of the movement of the cells inside the plant.^41^ There are many such machines, and the one used in this study was a 3D clinostat. We aimed to optimize a 3D clinostat for a life science experiment. Since a 3D clinostat is prepared with two motors, we fixed the angular velocity of one (primary) motor and varied for the angular velocity of the other (secondary) motor. In this setup, each motor ran continuously and constantly in one direction through the experiment; this device was used to rotate plants in three dimensions at a speed of 4 rotations/min.^42^ Wheatgrass seeds were germinated under these conditions for 7 days.^14^

### Preparation of plant extracts

To limit microbial growth, all equipment and containers involved in the germination process were treated with boiling water. Prior to germination, wheat grains of the same size were submerged in 1.25% sodium hypochlorite at room temperature for 30 min to disinfect and kill any contaminating microorganisms on the grain surface. Distilled water at ~10 °C was then run over the grains for 15 min to thoroughly rinse off any remaining sodium hypochlorite.^16^ The sterilized grains were steeped in tap water for 24 h at 21 °C. For germination, wheat grains were placed into a special container with drain holes at the bottom and irrigated with Hoagland’s solution. Germination was carried out in a plant growth room at 21 °C for 7 days. After harvest, the whole plants were washed carefully with distilled water for 3 min to remove any remaining perlite particles or nutrient elements.^16^

WGG and WGM conditions were dried under shade for 7 days at room temperature ranging from 29 to 32 °C with a relative humidity of 70–90% and then ground using a blender, while nongerminated seeds were discarded. An appropriate amount of the powdered plant material was extracted with ethanol using a Soxhlet extraction apparatus. The solvent was completely removed under reduced pressure in a rotary vacuum evaporator to yield the crude extract. All extracts were packed in an air-proof container before being stored at −20 °C until use.^43^

### Determination of the TP, flavonoid, and vitamin C contents

The TP content of WGG and WGM conditions was estimated using the Folin–Ciocalteu assay.^44^ A total of 475 µl of a 5% sodium carbonate solution was mixed with 50 µl of plant extract. The reaction mixture was incubated for 3–5 min, and 475 µl of 50% Folin–Ciocalteu reagent was added. The reaction mixture was mixed and incubated at room temperature in darkness for 1 h. The absorbance of the reaction product was measured at 724 nm using a ultraviolet–visible spectrophotometer. The TP content was calculated from a standard calibration curve based on gallic acid, and the results are expressed as mmol of gallic acid equivalents (GAEs) per 100 mg of plant dry weight (mmol GAE 100 mg^−1^ DW).

The total flavonoid (TF) content of WGG and WGM conditions was determined according to Karadeniz et al.^45^ Fifty microliters of plant extract and 600 µl of ddH_2_O were mixed with 40 µl of 5% potassium nitrite. The mixture was incubated at room temperature for 6 min, and then 70 µl of a 4.26% aluminum chloride solution was added. After 5 min of incubation at 25 °C, we added 240 µl of sodium hydroxide (1 M) to the reaction mixture, and the solution was mixed well. The absorbance of the reaction product was measured at 510 nm, and the TF content was calculated from a standard curve based on rutin. The results are expressed as mmol of rutin equivalents (REs) per 100 mg of plant dry weight (mmol RE 100 mg^−1^ DW).

Five milliliters of dilute extracts of *T. aestivum* germinated under gravity and microgravity conditions was added into a test tube, following which 1 ml of glacial acetic acid was added, the solution was titrated with a 2,6-dichlorophenolindophenol solution (0.2 g/l) until a faint permanent pink color was observed, after which the titer (T) was recorded.^46^ The titration was repeated with 5 ml of water as a blank (B 1) and 5 ml of an ascorbic acid standard solution (0.1 g/l). The vitamin C content of the test sample was calculated according to the following equation:

Vitamin C content (mg/dl) = (T − B1/ ST − B1) × 2 × dilution factor.

### Hydrogen peroxide- and nitric oxide-scavenging activity assays

The hydrogen peroxide-scavenging activity of ethanolic extracts of WGG and WGM conditions was evaluated according to the method of Ruch et al.^47^ The extract at different concentrations (3.4 ml, pH 7.4) was mixed with a hydrogen peroxide solution (43 mM, 0.6 ml, pH 7.4). After 10 min, the absorbance of the reaction mixture was measured at 230 nm. The reaction mixture without sample was used as a blank. Ascorbic acid was used as a reference compound. The percentage inhibition activity was evaluated as [(Abs. of the control − Abs. of the sample)/Abs. of the control] × 100%.

Nitric oxide was produced from sodium nitroprusside and measured by the Griess reaction.^48^ Nitric oxide was generated spontaneously from sodium nitroprusside compound dissolved in aqueous solution at physiological pH and interacted with oxygen to generate nitrite ions, which were measured by the use of Greiss reagent. Different concentrations of ethanolic extracts of WGG and WGM conditions dissolved in dimethyl sulfoxide were mixed with sodium nitroprusside (5 mM) in phosphate-buffered distilled water and incubated at 25 °C for 150 min. A control experiment without the test compound but with the equivalent amount of alcohol was conducted similarly. At intervals, samples (0.5 ml) of the incubation solution were diluted with 0.5 ml of Griess reagent (1% sulfanilamide, 0.1% naphthyl ethylenediamine dihydrochloride and 2% H_3_PO_4_). The absorbance of the chromophore was measured at 546 nm, and nitric oxide-scavenging activity was determined based on the absorbance of potassium as a standard solution.

### Induction of diabetes by STZ in rats

A total of 35 male albino Wistar rats (150–200 g body weight) were obtained from the animal facility at the University of Jordan. The animals were maintained under a constant 12-h light and 12-h dark cycle at 21–23 °C. In this study, animals were handled according to the guidelines of the American University of Madaba for the Care and Use of Laboratory Animals. The protocol used in this study was approved by the Ethics Committee of the American University of Madaba (5/2018).

Animals were fasted for 12 h, and diabetes mellitus was then induced in the rats by the intraperitoneal injection of freshly prepared STZ at a dose of 55 mg/kg in a 0.1 M citrate buffer solution at pH 4.5. The STZ-treated animals were allowed to drink a solution of 5% glucose dissolved in distilled water overnight to overcome drug-induced hypoglycemia. Three days after the STZ injection, blood was withdrawn from the tail vein, and the glucose level was determined immediately after the blood draw. Rats were considered diabetic when their fasting blood glucose levels were more than 250 mg/dl.

### Experimental design

After the induction of diabetes, the diabetic rats were randomly allocated to four groups (seven rats each): (1) a diabetic group that received 1% v/v Tween-80 in distilled water given orally, (2) a diabetic group that received 150 mg/kg bw ethanolic extract of WGM dissolved in 1% v/v Tween-80 in distilled water given orally, (3) a diabetic group that received 150 mg/kg bw ethanolic extract of WGG dissolved in 1% v/v Tween-80 in distilled water given orally and (4) a diabetic group that received 100 mg/kg bw metformin dissolved in 1% v/v Tween-80 in distilled water given orally. Seven rats received 1% v/v Tween-80 in distilled water orally as a vehicle and were used as a control group. The vehicle, metformin, and wheatgrass germinated extract were given orally by gavage as single daily treatments for 30 days. Blood was collected from the tail vein of the rats after overnight fasting on the 0th (before the start of the experiment), 3rd, 7th, 14th, 21st, and 30th day, and glucose levels were measured by using an Accu-Chek active glucometer.

### Oral glucose tolerance test

Briefly, after overnight fasting, the rats were intragastrically administered glucose (2 g/kg). Blood samples were withdrawn from the tail vein after 0, 30, 60, 90, and 120 min, and blood glucose levels were determined using an Accu-Chek active glucometer.

### Assay of biochemical parameters

After 30 days of treatment, the rats were fasted overnight and sacrificed. Blood was collected using EDTA as the anticoagulant, and whole blood was used to measure HbA1c, urea, and creatinine levels. The plasma was separated by collecting blood in a heparin-coated tube and centrifuging it at 1000 × *g* for 15 min at 4 °C. The plasma was used to estimate insulin, and plasma C-peptide assays were performed using a radioimmunoassay (RIA) kit for rats. TC, TG, HDL-C, and LDL levels and the activities of the liver enzymes AST and ALT were determined spectrophotometrically using commercial kits.

### Histological analysis

Specimens of the pancreatic tissue of the different groups were immediately fixed in 10% formalin and then underwent standard treatment with an alcohol gradient and xylene. For histopathological examination, 6-µm-thick pancreatic specimens were stained with hematoxylin and eosin. The β cells were counted under a ×40 objective lens. Cell densities are expressed as cells per pancreatic islet.^49^

### Statistical analysis

Statistical analysis was performed using GraphPad Prism 7. Values are expressed as the mean ± SEM, and data were analyzed by using two-way ANOVA, followed by Tukey’s multiple comparison test, with the level of significance set at *P* < 0.05.

## Supplementary information

nr-reporting-summary

## Data Availability

The authors declare that the data that support the findings of this study are available within the paper.
